# Computational Exploration of Phenolic Compounds in Corrosion Inhibition: A Case Study of Hydroxytyrosol and Tyrosol

**DOI:** 10.3390/ma16186159

**Published:** 2023-09-11

**Authors:** Hassane Lgaz, Han-seung Lee

**Affiliations:** 1 Innovative Durable Building and Infrastructure Research Center, Center for Creative Convergence Education, Hanyang University ERICA, 55 Hanyangdaehak-ro, Sangrok-gu, Ansan-si 15588, Gyeonggi-do, Republic of Korea; hlgaz@hanyang.ac.kr; 2 Department of Architectural Engineering, Hanyang University ERICA, 55 Hanyangdaehak-ro, Sangrok-gu, Ansan-si 15588, Gyeonggi-do, Republic of Korea

**Keywords:** corrosion inhibition, hydroxytyrosol, tyrosol, phenolic compounds, molecular dynamics simulation, density-functional tight-binding, quantum chemical calculation, adsorption characteristics, green inhibitor

## Abstract

The corrosion of materials remains a critical challenge with significant economic and infrastructural impacts. A comprehensive understanding of adsorption characteristics of phytochemicals can facilitate the effective design of high-performing environmentally friendly inhibitors. This study conducted a computational exploration of hydroxytyrosol (HTR) and tyrosol (TRS) (potent phenolic compounds found in olive leaf extracts), focusing on their adsorption and reactivity on iron surfaces. Utilizing self-consistent-charge density-functional tight-binding (SCC-DFTB) simulations, molecular dynamics (MD) simulations, and quantum chemical calculations (QCCs), we investigated the molecules’ structural and electronic attributes and interactions with iron surfaces. The SCC-DFTB results highlighted that HTR and TRS coordinated with iron atoms when adsorbed individually, but only HTR maintained bonding when adsorbed alongside TRS. At their individual adsorption, HTR and TRS had interaction energies of −1.874 and −1.598 eV, which became more negative when put together (−1.976 eV). The MD simulations revealed parallel adsorption under aqueous and vacuum conditions, with HTR demonstrating higher adsorption energy. The analysis of quantum chemical parameters, including global and local reactivity descriptors, offered crucial insights into molecular reactivity, stability, and interaction-prone atomic sites. QCCs revealed that the fraction of transferred electron ∆N aligned with SCC-DFTB results, while other parameters of purely isolated molecules failed to predict the same. These findings pave the way for potential advancements in anticorrosion strategies leveraging phenolic compounds.

## 1. Introduction

Owing to its rich organic compound composition, olive oil extract, and particularly olive leaf extract, has gained recognition in corrosion prevention [1,2,3]. The corrosion of materials, particularly metals and alloys, poses significant challenges in various industrial sectors. These challenges encompass not only the degradation of mechanical integrity but also the induction of substantial economic costs related to maintenance, replacements, and loss of functionality. Corrosion can lead to safety risks, especially in critical infrastructures such as bridges, pipelines, and aircraft [4]. Furthermore, traditional corrosion inhibitors have been associated with environmental toxicity and limited efficiency across varying corrosive environments, necessitating a quest for alternative, more versatile, and eco-friendly solutions [5,6].

Phytochemicals such as hydroxytyrosol and tyrosol, ubiquitously found in olive leaves, were chosen for investigation due to their renowned antioxidant and electronic properties [7,8,9,10,11,12]. Unlike other phytochemicals, the unique structural attributes of HTR and TRS, particularly the presence of hydroxyl groups, offer distinct potentials in anticorrosion applications. These phenolic compounds are characterized by electron-dense configurations and the abundance of active interaction sites within their structures, which could facilitate the formation of protective layers on metal surfaces [8,12,13,14,15]. The abundant availability, non-toxic nature, and existing empirical evidence of the olive leaf’s anticorrosive effects has prompted further exploration into their complex molecular interactions with iron surfaces.

When applied to surfaces of metals and alloys, these phytochemicals act synergistically to form a protective layer, thus impeding corrosive interactions between the metal surface and its environment [16,17]. Moreover, their eco-friendly and non-toxic features render these plant extracts a promising alternative to conventional synthetic corrosion inhibitors [18,19,20,21,22].

Several studies have demonstrated that olive leaf extracts exhibit exceptional anticorrosion properties due to their rich organic compounds, particularly hydroxytyrosol and tyrosol [1,3,15]. The proficiency of these phenolic compounds as corrosion inhibitors is ascribed to their unique structural characteristics, including multiple hydroxyl groups, heteroatoms such as O, N, S, and functional groups, known for promoting outstanding anticorrosion performance [23,24,25]. This research builds upon previous works by focusing on specific phenolic compounds, investigating their molecular mechanisms with iron surfaces, and exploring potential applications in corrosion prevention as isolated compounds, distinguishing the study from earlier research efforts [23,25,26,27].

Computational strategies that include quantum chemical computations, molecular dynamics simulations, and first-principles DFT simulations have been extensively used to assess inhibitors’ reactivity and adsorption characteristics [28]. These methodologies facilitate the detailed examination of individual compound reactivity, as well as their interactions with metallic surfaces. This approach presents a robust solution to understand the complexity inherent in molecular–surface interactions, providing valuable insights beyond what can be achieved through conventional experimental methods alone [29,30].

In one pioneering study, Nuha Wazzan conducted a comprehensive computational analysis of three phytochemical constituents from the Allium Jesdianum (AJ) flower using DFT and Monte Carlo simulations [31]. The author probed their potential as corrosion inhibitors, chemical reactivities, and adsorption propensities. The study involved a detailed exploration of local and global reactivity descriptors, such as the energies and distributions of the HOMOs, LUMOs, their gaps, and other reactivity parameters including hardness, global softness, global electronegativity, chemical potential, electrophilicity, nucleophilicity, and electron-accepting and -donating powers. Additionally, the author examined the adsorption behavior of these phytochemicals on three industrial metals—Fe(1 1 0), Al(1 1 1), and Cu(1 1 1)—revealing a predominant tendency for chemisorption. In another notable study, Kumar et al. investigated the corrosion inhibition properties of lawsone, gallic acid, and α-d-glucose—principal components of henna extract—on the Fe(001) surface, using the density functional theory [32]. Through the application of the density functional theory, the authors employed quantum chemical descriptors and interaction energies to forecast their relative efficiencies as corrosion inhibitors. Their findings supported the superiority of lawsone, followed by gallic acid and α-d-glucose. The authors reported that α-D glucose exhibited a weaker affinity to the surface, ascribed to steric effects preventing the interaction of its ring C and O with Fe-atoms on the surface.

Although the anticorrosive role of olive leaf extracts is well-documented empirically, a comprehensive understanding of the mechanistic aspects behind their exceptional performance remains largely unexplored. Computational investigations, akin to those conducted by other researchers on different plant extracts, can provide a detailed understanding of the adsorption characteristics and molecular features of their main components at the molecular level [31,32]. Molecular dynamics simulation is one such method that allows assessment of inhibitor–metal interactions in both vacuum and aqueous states [33,34,35]. Nonetheless, while useful in revealing the adsorption configuration and competitiveness of inhibitors, this methodology encounters limitations when attempting to simulate inherently quantum processes such as bond creation and dissolution during the adsorption of molecules onto metallic surfaces [36]. This limitation can be complemented using advanced atomistic modeling methods such as the SCC-DFTB approach (a computationally efficient variant of density functional theory (DFT)) [37,38,39,40,41]. The decision to utilize SCC-DFTB simulations stems from the need to study larger systems while retaining a reasonable level of accuracy. The SCC-DFTB method, unlike traditional DFT, allows for the examination of complex molecular structures and interactions without excessive computational demands. This approach provides a robust solution to understand the complexity inherent in molecular–surface interactions, offering insights into stable adsorption geometries, mechanisms of bond formation, and the electronic properties of the interacting entities, as well as the intensity of their adsorptive interactions [37,38,39,40,41]. Key parameters used to assess the strength of adsorption of the molecules onto the iron surface include quantifying the most energetically favorable adsorption geometries, the identification of optimal bonding configurations, and an examination of the interaction energies between the molecules and the metal surface [37,38,39,40,41].

In addition to these atomistic simulations, quantum chemical calculations have been employed to explore the reactivity of individual hydroxytyrosol and tyrosol molecules. The rationale for this approach stems from the ability of quantum chemical calculations to provide in-depth insights into the electronic properties, such as the energies and distributions of the frontier molecular orbitals, hardness, global softness, global electronegativity, chemical potential, electrophilicity, nucleophilicity, and electron-accepting and -donating powers [42]. Quantum chemical calculations facilitate a nuanced exploration of these aspects, going beyond what is achievable through simulation techniques.

Furthermore, these quantum chemical calculations complement the atomistic simulations by providing a more detailed and specialized layer of analysis. While atomistic simulations such as SCC-DFTB provide valuable insights into stable adsorption geometries and the overall interaction between molecules and surfaces, quantum chemical calculations allow for a closer examination of the specific electronic and molecular characteristics that could govern these interactions. By coupling these two approaches, the study benefits from both a macroscopic view of molecular behavior and a microscopic understanding of the underlying electronic processes, offering a comprehensive and multidimensional view of the reactivity profile of the molecules.

In light of this, and in the absence of a comprehensive study encapsulating both electronic and reactivity features as well as the underlying bonding mechanisms of hydroxytyrosol and tyrosol on the Fe(110) surface, herein, a computational examination of these phenolic compounds was carried out. Semi-empirical SCC-DFTB simulations, molecular dynamics (MD) simulations, and quantum chemical calculations (QCCs) were used to explore the adsorption characteristics of hydroxytyrosol and tyrosol at their isolated and adsorbed states. Insights into the adsorption configurations of these molecules on the iron surface, both in the absence and presence of water molecules, were derived from the molecular dynamics (MD) simulations. Additionally, interactions between the molecules and iron were subjected to SCC-DFTB simulations with the intent of identifying the most energetically favorable adsorption geometries and quantifying the associated interaction energies of the two compounds, both individually and synergistically, on the Fe(110) surface. The findings from this study offer interesting prospects for further development and valorization of the compounds under investigation.

## 2. Computational Details

### 2.1. Quantum Chemical Calculations

The exploration of the reactivity of individual hydroxytyrosol and tyrosol molecules necessitates the execution of quantum chemical calculations. The geometry of the compounds under test was fully optimized using DFT along GGA functional and DNP basis sets [43], employing DMol3 code implemented in Materials Studio [44,45]. The relationships below, as previously outlined in the existing literature [46], were used to compute these quantum chemical reactivity indices:

(1) $$\mathrm{Energy}\;\mathrm{gap}:\;\Delta {\mathrm{Eg}=E}_{\mathrm{LUMO}}{\;-\;E}_{\mathrm{HOMO}}$$

(2) $$\mathrm{Electronegativity}\;\mathrm{and}\;\mathrm{chemical}\;\mathrm{potential}:\;\chi =-\;\mu =-\frac{E_{\mathrm{LUMO}}+E_{\mathrm{HOMO}}}{2}$$

(3) $$\mathrm{Hardness}:\;\eta =\frac{E_{\mathrm{LUMO}}-E_{\mathrm{HOMO}}}{2}$$

(4) $$\mathrm{Softness}:\;\sigma =\frac{1}{\eta }$$

(5) $$\mathrm{Electrophilicity}\;\mathrm{index}:\;\omega =\frac{\mu^{2}}{2\eta }$$

(6) $$\mathrm{Nucleophilicity}\;\mathrm{index}:\;\varepsilon =\frac{1}{\omega }$$

(7) $$\mathrm{Fraction}\;\mathrm{of}\;\mathrm{transferred}\;\mathrm{electron}:\;\Delta N=\frac{\chi_{\mathrm{metal}}-\chi_{\mathrm{mol}}}{2\left(\eta_{\mathrm{metal}}+\eta_{\mathrm{mol}}\right)}=\frac{\Phi -\chi_{\mathrm{mol}}}{2\eta_{\mathrm{mol}}}$$

where $\Phi_{Fe}$ is set at 4.82 eV and the total hardness $\eta_{Fe}$ of iron is assigned a value of zero.

Hirshfeld population analysis was used to examine the Fukui function indices following the equations below [47]:

(8) $$f_{k}^{+}=q_{k}\left(N+1\right)-q_{k}\left(N\right)$$

(9) $$f_{k}^{-}=q_{k}\left(N\right)-q_{k}\left(N-1\right)$$

(10) $$\Delta f\left(k\right)=f_{k}^{+}-f_{k}^{-}$$

where $q_{k}\left(N+1\right)$, $q_{k}\left(N\right)$, and $q_{k}\left(N-1\right)$ correspond to the electron densities on atom *k* for systems with *N* + 1, *N*, and *N* − 1 electrons, respectively.

The molecular electrostatic potential (MEP) maps were calculated using DFT, the B3LYP functional, and a 6-311G basis set, employing Gaussian Ver. 09W software [48]. The visualization of these maps was carried out using GaussView 6.0 [49].

### 2.2. Molecular Dynamics Simulation

The use of molecular dynamics (MD) simulation is helpful in extracting information on the adsorption configurations and magnitude of the interaction between investigated molecules and the iron surface in a simulated solution environment. To this end, a (110)-oriented iron unit cell was transformed into a (7 × 7) supercell, having 6 iron layers and attached to a vacuum layer or a solvent layer composed of a single molecule and 500 water molecules to simulate the interaction between HTR and TRS on the Fe(110) surface in vacuum and aqueous states. The resulting simulation box had the following size: 19.86 × 19.86 × 35.48 Å^3^. The initial optimization of the designed simulation boxes was performed using the smart minimizer protocol, followed by a 5000 picosecond-long MD equilibrium process within the canonical ensemble (NVT) employing the COMPASSIII force field [50]. The electrostatic and van der Waals non-bonded interactions were addressed using Ewald summation and atom-based cut-off techniques, respectively. The velocity Verlet integration scheme, with a time step of 1 femtosecond, was implemented to solve Newton’s equation of motion [51]. A Nosé–Hoover thermostat set at 303K [52] was used to control the temperature. All molecular dynamics simulations were performed utilizing the Forcite module integrated within the Materials Studio software ver. 6.0 [53]. The adsorption energies of the molecules on the Fe(110) surface were derived from these MD simulations via single-point energy calculations for the complex adsorption system ($E_{\mathrm{total}}$), the metal surface in the absence and presence of solution ($E_{\mathrm{surface}};\;E_{\mathrm{surface}+\mathrm{solution}}$), the inhibitor molecule both with and without the solution ($E_{\mathrm{molecule}};\;E_{\mathrm{molecule}+\mathrm{solution}}$), and the energy of the solution $\left(E_{\mathrm{solution}}\right)$ as follows:

(11) $$\mathrm{Vacuum}\;\mathrm{state}:\;E_{\mathrm{ads}}=E_{\mathrm{total}}-\left(E_{\mathrm{surface}}+E_{\mathrm{molecule}}\right)$$

(12) $$\mathrm{Aqueous}\;\mathrm{state}:\;E_{\mathrm{ads}}=E_{\mathrm{total}}-\left(E_{\mathrm{surface}+\mathrm{solution}}+E_{\mathrm{molecule}+\mathrm{solution}}\right)+E_{\mathrm{solution}}$$

### 2.3. SCC-DFTB Computational Details

**Figure 1:**
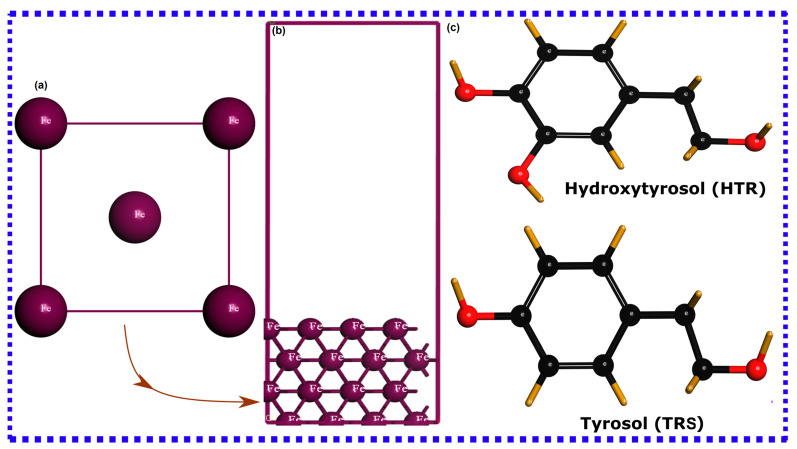
(**a**) Iron crystal structure, (**b**) DFTB-simulated box, and (**c**) molecular structure of HTR and TRS molecules.

The SCC-DFTB technique, which is a computational method based on the second-order expansion of the Kohn–Sham density functional theory was used for atomistic simulations. The suitability of the SCC-DFTB methodology in simulating organic transition metal adsorption systems is well-documented in earlier research [37,54,55,56]. This method offers a substantial advantage in terms of predicting electronic and structural properties with excellent accuracy as first-principle DFT simulations, but at a significantly increased speed, particularly for larger systems. In the current investigation, the spin-polarized SCC-DFTB method was employed to fully optimize the interaction between hydroxytyrosol and tyrosol molecules and the iron, counting for dispersion interactions with Slater–Koster trans3d parameters implemented in DFTB+ code [57]. The adsorption systems underwent a comprehensive optimization process, employing an SCC tolerance of 10^−8^ atomic units, thermal smearing (via the Methfessel–Paxton smearing distribution function), and the Broyden mixing scheme. All remaining convergence tolerance metrics were configured to align with fine quality standards. In parallel, the refinement of bulk lattice parameters was conducted using an (8 × 8 × 8) k-point grid. The optimization process yielded a lattice parameter value of 2.871 Å, impressively close to the experimental value of 2.862 Å, thus affirming the appropriateness of the chosen parameters. For the optimization of molecule Fe(110) systems, convergence was achieved using a (2 × 2 × 1) k-point grid. Adsorption models were established by constructing the Fe(110) iron surface based on a (3 × 3) supercell, separated by a 20 Å vacuum layer along the z-axis to prevent interactions between periodic images in all directions. The resulting simulation box had the following size: 9.92 × 9.92 × 26.08 Å^3^. The utilized iron crystal, the simulation box, and the molecular structures of hydroxytyrosol and tyrosol are depicted in Figure 1. Molecules were positioned, individually and synergistically, on the slab’s top layer and all atomic particles, apart from the two lowest atomic layers, were given the freedom to relax. Simulations were then conducted with surface coverages of 1/9 and 2/9 ML for individual and synergistic adsorption, respectively.

The optimization of standalone molecules via SCC-DFTB was achieved by creating a cubic box measuring 30 Å. The calculation of interaction energies was performed by evaluating the difference in the total energy of the adsorbed system and the sum of the isolated energies of the components. This allowed for a precise assessment of the relative strength of molecular adsorption on the Fe(110) surface using the following formula:

(13) $$E_{inter}=E_{mol/surf}-\left(E_{mol}+E_{surf}\right)$$

where $E_{mol}$, $E_{surf}$, and $E_{mol/surf}$ denote the total energies of the standalone molecules, the Fe(110) iron surface, and the combined molecule/Fe(110) adsorption systems, respectively.

## 3. Results and Discussion

### 3.1. Quantum Chemical Calculations

#### 3.1.1. Global Reactivity Descriptors

Quantum chemical calculations could serve as a crucial tool, enabling the enhancement of our scientific understanding of the overall reactivity of molecules under investigation. A variety of global reactivity indicators, including the HOMO, the LUMO, the energy gap, ionization energy, electron affinity, and the fraction of transferred electrons, are used to evaluate the reactivity of inhibitor molecules. This allows a better understanding of their electronic attributes in the absence of any external influence [31].

**Figure 2:**
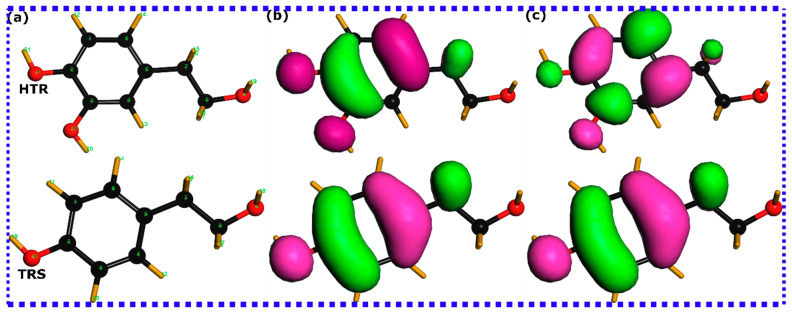
DFT-refined structures of HTR and TRS molecules as derived through the DFT/GGA approach in panels (**a**). Visualizations of the HOMO and the LUMO are correspondingly represented in panels (**b**) and (**c**), respectively.

Before delving into the detailed numerical analysis of the quantum chemical parameters listed in Table 1, it could be insightful to initially examine the iso-surface maps for both HOMOs and LUMOs of HTR and TRS molecules. These visual representations are demonstrated in Figure 2. The HOMO density distribution on a molecule signifies an electron-rich area that serves as the center for chemical interactions through electron donation [42]. Conversely, the LUMO density marks a region on the molecule that has the ability to accept electrons [42]. Investigating the HOMO/LUMO maps for HTR and TRS molecules suggests that HOMO and LUMO densities are dispersed across the complete molecular structures, except the hydroxymethyl group. This indicates that the whole molecular structures may act as either electron donors or acceptors when they interact with the metal surface. Notwithstanding, considering the improbability of a molecule engaging in donor–acceptor interactions throughout all its atomic sites, the HOMO/LUMO mappings fall short in providing a definitive distinction of the reactivity among the investigated molecules. Nonetheless, the reinterpretation of these maps in terms of energy may potentially enable a more comparative assessment. It is generally assumed that a molecule with higher HOMO energy (E_HOMO_) exhibits an increased capacity for electron donation, while a molecule with lower LUMO energy (E_LUMO_) demonstrates a high tendency to accept electrons [31]. An examination of the quantum chemical parameters listed in Table 1 indicates that the HTR molecule slightly outperforms the TRS molecule in terms of electron donation ability, as its HOMO energy is −6.22 eV, while that of TRS is −6.733 eV. In contrast, the TRS molecule exhibits a significantly stronger electron acceptance tendency compared with HTR, given its lower LUMO energy (−6.355 eV) compared with that of HTR (−2.116 eV).

Chemical hardness (η) and softness (σ) are critical parameters in evaluating the stability and reactivity of a molecule. Chemical hardness is a measure of the resistance of a molecule to changes in electron distribution and is related to the stability and reactivity of the molecule. A higher hardness value implies lower reactivity, as the molecule is less likely to participate in charge transfer with other species [58,59]. Chemical softness is the inverse of hardness; softness quantifies a molecule’s readiness to engage in chemical reactions by accepting or donating electrons. Higher softness indicates a greater tendency for the molecule to interact with other species [58,59]. A molecule with substantial chemical hardness, hence a significant energy gap, necessitates a high amount of energy to undergo chemical transformations or interact with other molecules, attributed to the large energy barrier impeding electron transfer [60]. In contrast, a molecule with high chemical softness, the reciprocal of hardness, indicates a higher propensity for electron transfer processes with other entities, such as during nucleophilic attacks [60]. The data listed in Table 1 suggest that the TRS molecule exhibits a very small energy gap value (0.378 eV) compared with the HTR molecule (4.104 eV); it can thus be inferred that the TRS molecule would exhibit greater softness compared with the HTR molecule, indicative of a decrease in stability and an increase in reactivity in its isolated state.

The concepts of nucleophilicity (ε) and electrophilicity (ω) signify the capacity of an electron-rich molecule to donate electrons and an electron-deficient molecule to receive electrons, respectively [60]. While conceptual similarities exist, these parameters are not strictly correlative with HOMO and LUMO energies. Data represented in Table 1 reveal that the TRS molecule exhibits significantly higher electrophilicity in comparison to the HTR molecule, while an inverse pattern is discernible concerning nucleophilicity.

The reactivity descriptor known as the fraction of electrons transferred (ΔN) offers valuable insights into the tendency of molecules to transfer their electrons to a metal surface [61,62]. Contrary to other parameters, this descriptor holds significant importance within the context of corrosion inhibitors as it elucidates the molecule’s interaction capabilities with a metal surface [63,64]. Previous research demonstrated that a positive ΔN value signifies the molecule’s potential to transfer its electrons, while a negative value indicates the inverse [61,62]. In this study, the derived ΔN values suggest that the HTR molecule exhibits a higher tendency to transfer its electrons to the iron surface, whereas the TRS molecule demonstrates the opposite behavior. The ΔN value of TRS (−4.56) is very negative compared with the positive ΔN value of HTR (0.158). This suggests that this parameter might accurately predict the adsorption strength and bonding of the molecules under investigation with the iron surface.

#### 3.1.2. Fukui Function Indices

In relation to local reactivity descriptors, it is crucial to note that the process of corrosion inhibition, predominantly governed by electron transfer controlled reactions, necessitates an in-depth exploration of molecular local reactivity [65,66,67]. Such an investigation could provide critical insights into their interaction patterns with iron atoms. An efficient example of these local reactivity descriptors are Fukui functions, as put forth by Kenichi Fukui [68]. This theory elucidates the behavior of electrons in a molecule when subjected to slight perturbations [65]. Accordingly, Fukui functions can designate which atoms or regions in a molecule will function as nucleophilic (with electron-donating tendencies) or electrophilic (with electron-accepting tendencies) sites [66,67]. These indices enhance our understanding of the local reactivity of hydroxytyrosol (HTR) and tyrosol (TRS) by identifying specific regions in the molecules that are prone to either donate or accept electrons. This helps in predicting how these molecules will interact with other substances or surfaces. The clear identification of nucleophilic and electrophilic sites allows for an anticipatory model of how these compounds might bond with other molecules or metal surfaces, thus enhancing the understanding of their chemical behavior. Additionally, this understanding can be applied to predict potential interaction sites on other molecules, guiding the design and optimization of new compounds with desired properties.

**Figure 3:**
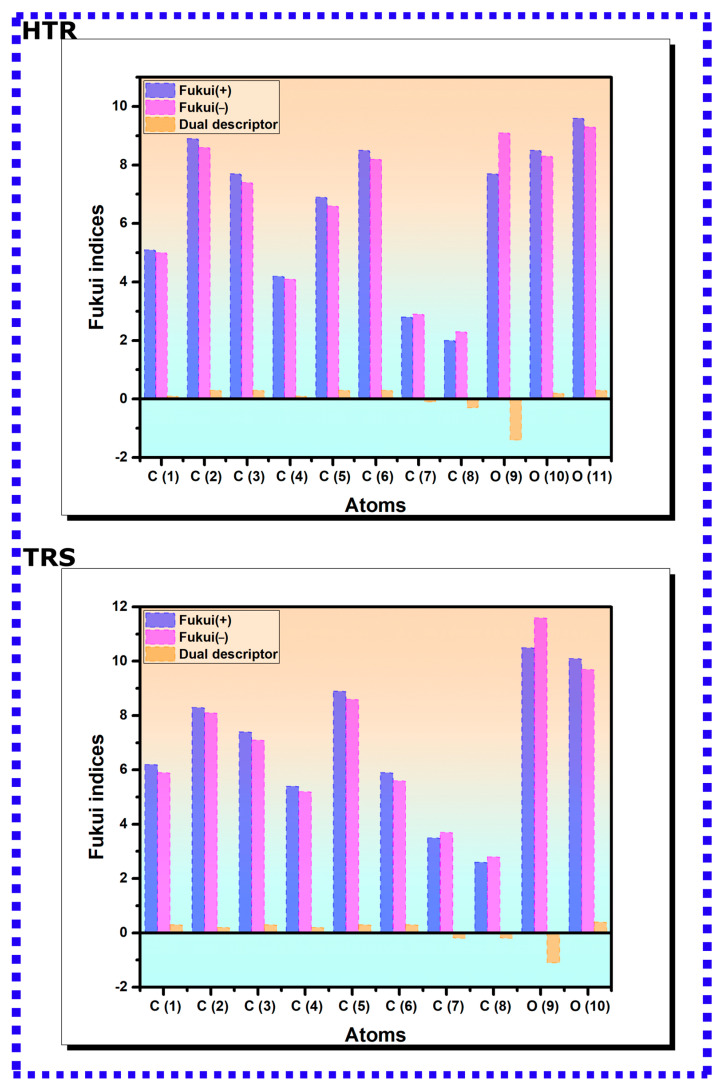
Depiction of Fukui function and dual descriptor values associated with HTR and TRS molecules as determined by the DFT/GGA technique.

Fukui function indices of HTR and TRS molecules are categorized in Figure 3. A high $f_{k}^{+}$ at a site signifies pronounced electrophilic properties (vulnerability to nucleophilic attacks), while the highest $f_{k}^{-}$ conveys a high nucleophilic character (susceptibility to electrophilic attacks) [47]. In addition, Morell et al. proposed a reactivity descriptor known as the dual descriptor to accurately depict the chemical reactivity of molecules, given that an atomic site can act both as a nucleophile and electrophile concurrently [69]. The results displayed in Figure 3 suggest that all atoms of both molecules exhibit high electrophilic and nucleophilic properties. In the case of HTR and TRS, the O(11) and O(9) atoms exhibit the highest electrophilic and nucleophilic characteristics, respectively. Regarding the dual descriptor, when the dual descriptor value is positive, the particular atom within a molecule is more prone to a nucleophilic attack, suggesting the atom is a favorable site for the donation of an electron [69]. On the contrary, when the dual descriptor value is negative, the atom is more susceptible to an electrophilic attack, indicating the atom is a suitable site for the acceptance of an electron [69]. From Figure 3, it can be noticed that the O(9) atom exhibits a significantly negative dual descriptor value in both HTR and TRS molecules, indicative of its strong electron-accepting ability when interacting with external species. Additionally, it is noticed that the additional hydroxy group in HTR exhibits high electrophilic and nucleophilic characters, which could enhance the affinity of HTR molecule to iron atoms as it presents an additional reactive site.

**Figure 4:**
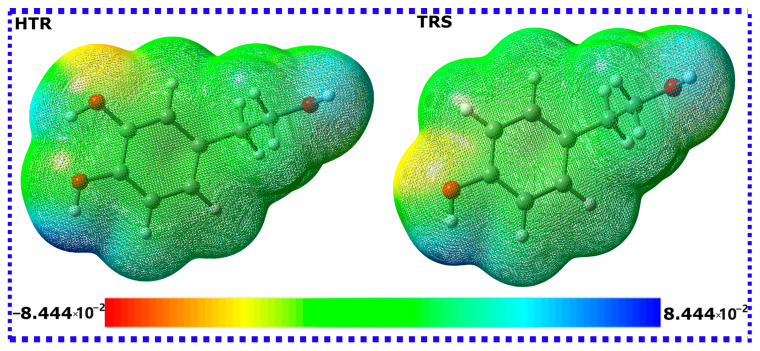
MESP of HTR and TRS molecules obtained by DFT/B3LYB method.

The molecular electrostatic potential (MEP) provides a valuable graphical tool, critical in the elucidation of molecular reactivity [70]. Within this method, the electron density is presented in a color-graded contour, offering a definitive depiction of polarization effects within the molecule. Areas characterized by high electron density are marked by hues of red or orange, while regions with low electron density are signified by shades of blue [71]. In the context of corrosion inhibition research, such MEP maps can be considered as graphical indicators of the electrophilic and nucleophilic characteristics of molecules. As depicted in Figure 4, it is evident that both HTR and TRS molecules show a red/orange region on their hydroxy groups attached to the phenyl ring. In contrast, the hydroxymethyl group displays a blue color in both molecules. The zones of high electron density (hydroxy groups) in both molecules can act as binding points with the iron surface, thereby contributing to their corrosion inhibition capabilities [72].

### 3.2. Semi-Empirical SCC-DFTB Calculations

#### 3.2.1. Optimized Geometries and Interaction Energies

**Figure 5:**
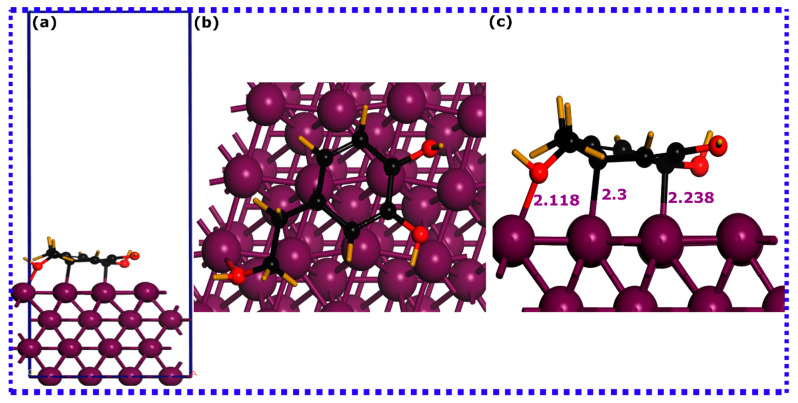
SCC-DFTB optimized adsorption structures of the hydroxytyrosol (HTR) molecule on the Fe(110) surface: (**a**) lateral perspective, (**b**) superior perspective, and (**c**) magnified lateral view. Bond lengths are denoted in angstroms (Å).

**Figure 6:**
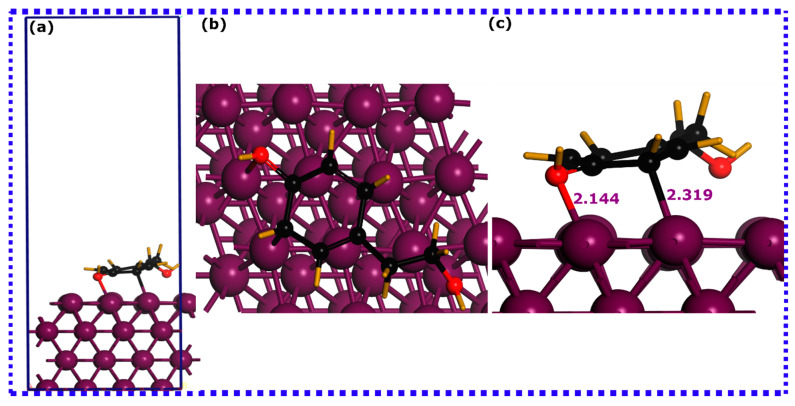
SCC-DFTB optimized adsorption structures of the tyrosol (TRS) molecule on the Fe(110) surface: (**a**) lateral perspective, (**b**) superior perspective, and (**c**) magnified lateral view. Bond lengths are denoted in angstroms (Å).

**Figure 7:**
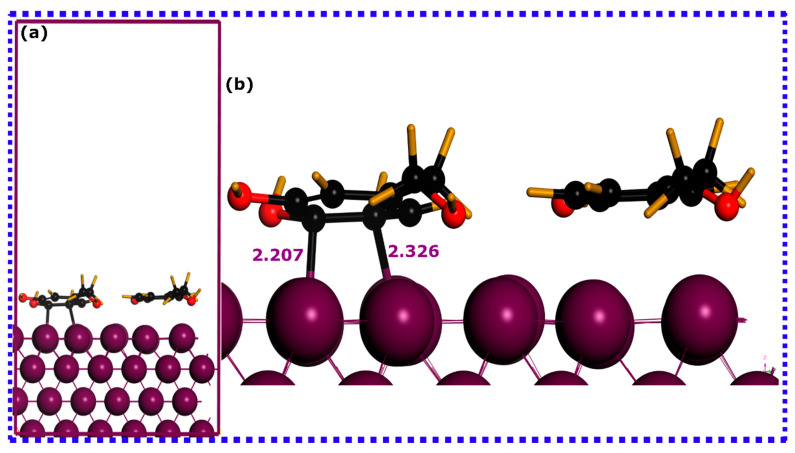
SCC-DFTB optimized adsorption structures of the hydroxytyrosol (HTR) and tyrosol (TRS) molecules on the Fe(110) surface: (**a**) lateral perspective, and (**b**) superior perspective. Bond lengths are denoted in angstroms (Å).

Utilizing SCC-DFTB simulations, an effective and reliable method for elucidating atomic scale behavior of molecule–metal adsorption systems, provides reliable, precise, and scientifically meaningful insights [41]. In this context, the adsorption process and interactions of HTR and TRS molecules onto the iron surfaces were assessed using DFTB simulations. Figure 5, Figure 6 and Figure 7 depict DFTB-optimized adsorption configurations of HTR, TRS, and both HTR/TRS on the Fe(110) surface.

It is a common fact that inhibitor molecules may bind to metal surfaces via complex physicochemical adsorption [73]. The sum of the covalent radii of atoms participating in interactions usually reflects the formation of a chemical bond if the interatomic distance is within this sum [74]. Conversely, physical interactions tend to emerge at interatomic distances exceeding 3 Å [74]. By inspecting the optimized adsorption configurations for HTR and TRS molecules, it is evident that their configuration on the iron surface is different. The HTR molecule is in close contact with the top layer of the Fe(110) surface (Figure 5), forming three bonds with its atoms. This molecule stabilizes in a parallel configuration to the surface through a single Fe-O and two Fe-C bonds. The Fe-O bond has a length of 2.118 Å, while the two Fe-C bonds have a distance between 2.3 and 2.238 Å. The TRS molecule, on the other hand (Figure 6), stabilizes nearly perpendicularly on the surface of the iron, forming Fe-O and Fe-C bonds with 2.144 Å and 2.319 Å lengths, respectively.

When considering synergistic adsorption (Figure 7), both molecules stabilize in a parallel disposition to the Fe(110) surface. However, while the HTR molecule coordinates with Fe atoms via two C atoms, the TRS molecule does not form any bond with the iron atoms. This indicates that, when both molecules interact synergistically on the iron surface, HTR would have a higher affinity to iron atoms, while TRS, in this scenario, appears to interact physically with the metal surface [75].

A better understanding of the bond nature formed between HTR and TRS molecules and iron atoms can be captured by correlating the bond distances with the sum of the covalent radii of interacting atoms [75]. Previous research has indicated that the cumulative covalent radii for Fe-C (r_C_ + r_Fe_) and Fe-O (r_O_ + r_Fe_) are 2.08 and 1.98 Å, respectively [76]. Upon comparing these with the computed bond distances, it becomes apparent that the lengths of Fe-C and Fe-O bonds fall within the sum of the corresponding covalent radii, suggesting potential chemical interactions between the molecules’ atoms and the iron surface.

The evaluation of interaction energies derived from optimized adsorption configurations can shed more light on the adsorption potency of HTR and TRS molecules on the iron surface. As indicated in the figure of each optimized structure, the HTR@Fe(110) exhibits a high interaction energy magnitude, suggesting its highest adsorption abilities, followed by TRS@Fe(110) having the lowest interaction energy magnitude, while synergistic adsorption exhibits the strongest interaction energy. HTR, TRS, and HTR/TRS systems exhibit negative interaction energies of −1.874, −1.598, and −1.976 eV, respectively, suggesting their thermodynamically preferred adsorption and propensity towards interactions with iron atoms [77,78]. These results highlight the excellent adsorption characteristics of the HTR compound compared with TRS, both individually and synergistically, as it has more tendency to coordinate with iron atoms in both situations. Given the findings, it can be inferred that hydroxy groups play a decisive role in molecule adsorption in their individual adsorption. However, when simulated together, the additional hydroxy group in HTR seems to strengthen its adsorption capacity, resulting in more favorable adsorption on the Fe(110) surface compared with the TRS molecule. Bearing these in mind, interactions governed by long-range dispersion forces can nonetheless exert a considerable influence on the interactions between molecules and surfaces, when TRS is adsorbed together with the HTR molecule, and might contribute to its interaction energy [79]. Experimental investigations mainly suggest complex interactions that involve both physical and chemical interactions, particularly in acidic mediums, both contributing to the stability of the formed inhibitor layer on the metal surface [80,81,82]. The examination of optimized adsorption configurations via projected density of states can potentially yield additional relevant data concerning the underlying bonding mechanisms.

#### 3.2.2. PDOS Analysis

**Figure 8:**
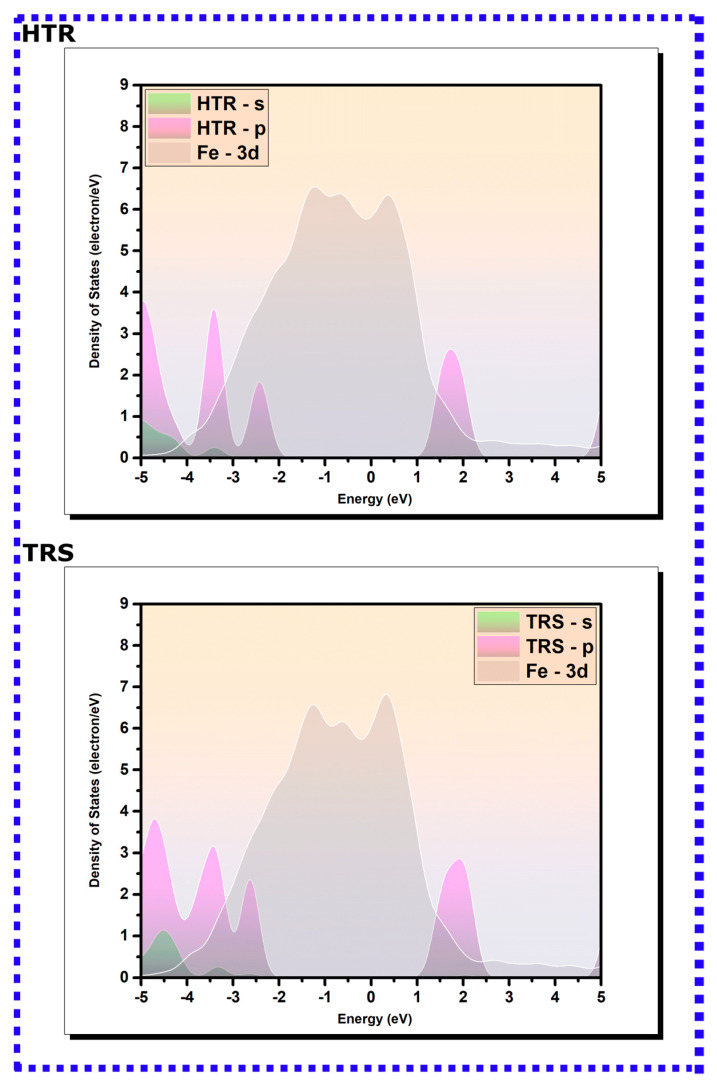
The PDOS for isolated HTR and TRS molecules on the Fe(110) surface, positioned 7 Å above the highest iron layer. The Fermi energy (EF) is selected as the reference point for the zero-energy level.

**Figure 9:**
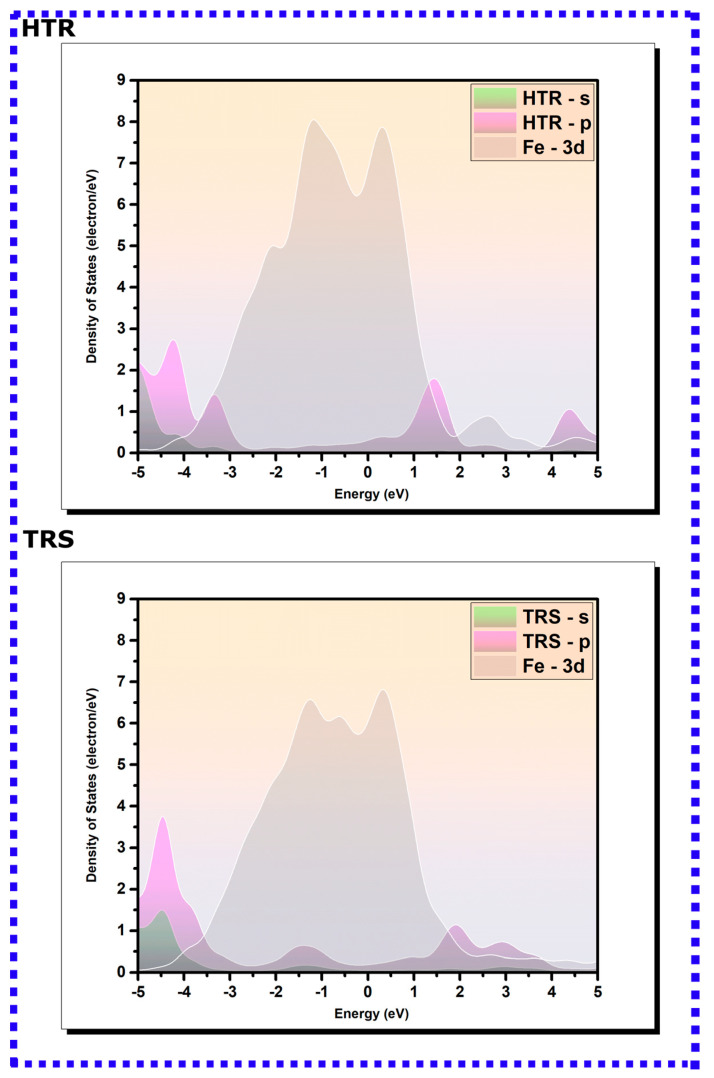
The PDOS for adsorbed HTR and TRS molecules on the Fe(110) surface. The Fermi energy (EF) is selected as the reference point for the zero-energy level.

The comprehensive elucidation of the electronic configuration of the optimized adsorption geometries of hydroxytyrosol (HTR) and tyrosol (TRS) on the Fe(110) surface can be obtained from the pictorial representation of the density of states, projected onto the s and p orbitals of the molecules as well as the 3d orbitals of iron [83,84,85]. Figure 8 presents the density of states’ plots of isolated molecules (positioned 7 Å above the topmost iron layer), whereas Figure 9 depicts the density of states for HTR and TRS following their adsorption onto the iron surface, alongside those corresponding to the 3d iron orbitals. The spectral modifications visible in the density of states’ profiles pre- and post-adsorption on the Fe(110) surface reflect the nature of their bonding interactions with iron atoms. As can be revealed from Figure 8, intense peaks manifest in the density of states’ plots for HTR and TRS molecules prior to adsorption. A significant fraction of these peaks aligns within the −5/5 eV energy interval, coinciding with the presence of the 3d bands of iron [32]. Such a scenario can promote the overlay and hybridization of the s and p orbitals of the molecules with the unoccupied d-orbitals of the iron atoms [39,40]. This alignment signifies hybridization, as the orbitals of the molecules and iron atoms interact in a manner that facilitates covalent coordination. The overlap between these orbitals allows for shared electrons to form bonding states, resulting in a more stable interaction between the molecules and the iron surface [39,40].

The PDOSs of adsorbed molecules display a noticeable broadening of molecular peaks compared with their initial pre-adsorption states, suggestive of an enhancement in molecular interactions. Simultaneously, a decrease in the intensity of these peaks is discerned. Moreover, a marked shift towards lower energy values is apparent in the post-adsorption state. These changes reflect the alterations in the electronic structure and energetics upon adsorption of HTR and TRS on the Fe(110) surface [85,86,87,88]. Specifically, the observed shift in energy values towards lower levels signifies a release of energy upon adsorption, often associated with a more stable state. The changes in peak intensity and distribution may point to the redistribution of electronic states and the formation of new bonding interactions between the molecules and the iron surface [32]. The molecular orbitals of the inhibitors are expected to hybridize with the metal’s d-states, creating hybrid orbitals via a charge transfer process, thus leading to notable modifications in the PDOS peaks accompanied by significant changes and redistribution within them [89,90]. Hence, it can be concluded that HTR and TRS covalently coordinate with the iron surface.

### 3.3. MD Simulations

**Figure 10:**
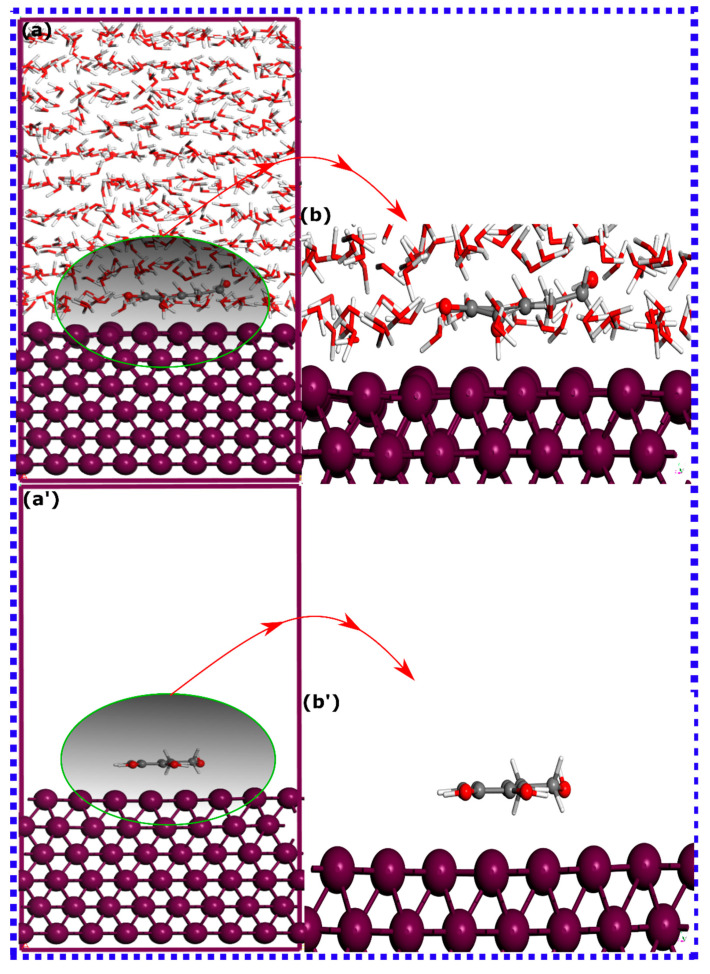
The most thermodynamically stable adsorption configurations for HTR molecules on the Fe(110) surface in (**a**) aqueous environment and (**a’**) vacuum, as derived from molecular dynamics (MD) simulations. (**b**,**b’**) are zoomed views of (**a**,**a**’).

**Figure 11:**
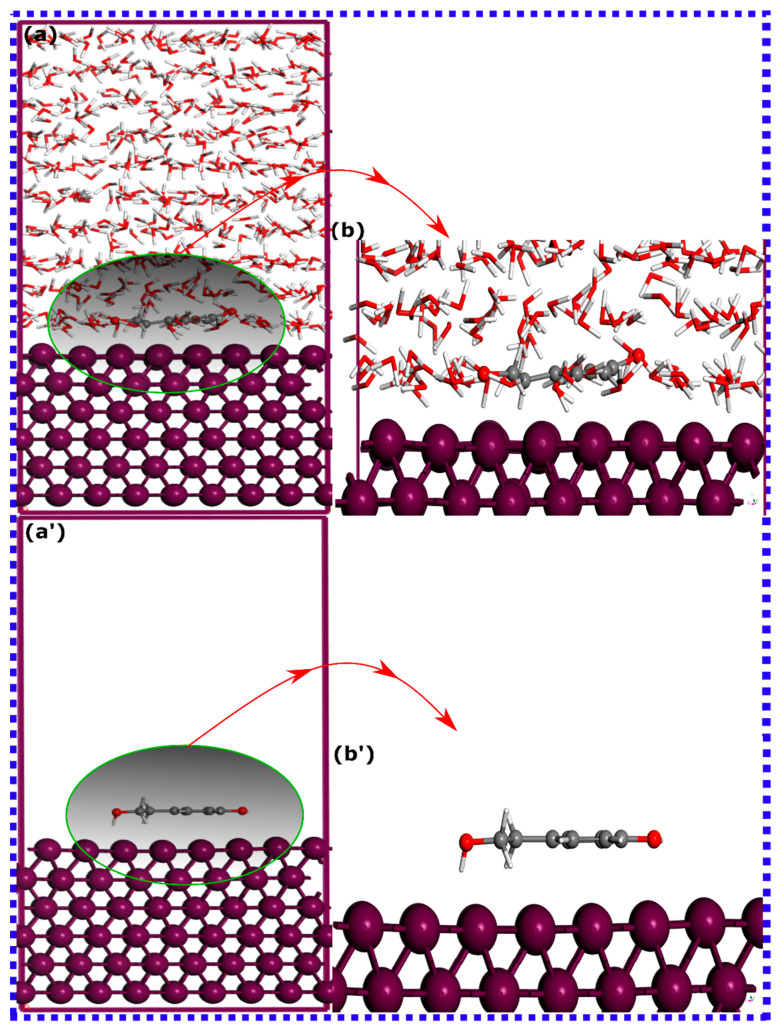
The most thermodynamically stable adsorption configurations for TRS molecules on the Fe(110) surface in (**a**) aqueous environment and (**a’**) vacuum, as derived from molecular dynamics (MD) simulations. (**b**,**b’**) are zoomed views of (**a**,**a’**).

Molecular dynamics (MD) simulation provides a powerful computational tool for investigating the microscopic processes involved in molecular adsorption onto metallic surfaces [91,92]. By simulating the intricate dynamics of atoms and molecules, MD simulations allow for the examination of the system at a more detailed level, enhancing our theoretical understanding of adsorption phenomena [28]. MD simulations can take into consideration all types of interactions within the system, including those between the molecules and the surface, as well as other potential influences. Assessing the trajectories of the simulated inhibitor–metal interactions, especially in a more explicit aqueous environment, can offer useful insights into the configuration and adsorption behavior of molecules. Herein, MD simulations are executed to determine the most energetically favorable adsorption configurations of HTR and TRS on the surface of iron at 303 K, with and without water molecules. The MD-optimized adsorption configurations for HTR and TRS molecules are demonstrated in Figure 10 and Figure 11, respectively.

The adsorption configuration suggests that both molecules, in both aqueous and vacuum states, predominantly adopt a disposition parallel to the iron surface. In this case, there could be a high possibility of an exchange of electrons, which may involve electron donation to the unoccupied orbitals of the metal. Even in an aqueous state, where the competition for adsorption is high, the adsorption configuration appears to remain constant, preserving the parallel orientation on the Fe(110) surface. Therefore, as molecules exhibit similar adsorption behavior, limited information can be obtained about the adsorption strength of each system without calculating their adsorption energies. It is vital to acknowledge that classical molecular dynamics simulations often lead to an inflated estimation of the adsorption energy between a molecule and a metallic surface. This inflated estimation can be traced back to the limitations of the employed force fields, which often neglect to account for phenomena such as electronic polarization effects and quantum mechanical perturbations [90,93]; nevertheless, it is still relevant for a comparative analysis. Table 2 presents the energetic analysis for both molecules on the Fe(110) surface. Predictably, the HTR molecule shows the strongest adsorption energy in both aqueous and vacuum states. The lower adsorption energies for TRS may be due to its decreased affinity towards the iron surface. However, both molecules have negative adsorption energies, indicative of their spontaneous and favorable adsorption on the iron surface [35]. Furthermore, the interaction energies of both molecules, when calculated in a vacuum state, register more negative values relative to those computed in the aqueous environment, as shown in Table 2. In a solvated state, molecules’ adsorption can be affected by water molecules because of hydrogen bond formation with the molecule, thus decreasing its interactive force. This can result in the reduction of the interactive force of molecules on the iron surface [35]. The findings from MD simulations appear to corroborate the conclusions derived from SCC-DFTB simulations.

Given the results obtained from molecular dynamics (MD) simulations, quantum chemical parameters, and SCC-DFTB simulations, a complex picture of the reactivity of the molecules in question emerges.

Electron Donating Abilities: It can be postulated that the reactivity of the molecules aligns well with their ability to donate electrons. The hydroxytyrosol (HTR) molecule’s less negative HOMO energy and higher ΔN value suggest a propensity to participate in electron exchange with electron-deficient iron atoms. This can lead to the formation of a bond between the molecule and the metal surface, influencing the overall interaction and adsorption characteristics.

Lack of Correlation with Quantum Chemical Parameters: The observation that quantum chemical parameters describing the reactivity of the molecules in a purely isolated state do not show any correlation with SCC-DFTB atomistic simulation is quite intriguing. This disjunction underscores the complexity of molecular behavior and suggests that a comprehensive understanding of reactivity requires a more nuanced approach.

Fraction of Transferred Electron: In contrast, the fraction of transferred electron, the only quantum chemical parameter associated with the metal surface, is found to align with the results of atomistic simulations. This highlights the importance of considering not just the intrinsic properties of the molecules but also the influence of the surrounding environment, such as the metal surface and solvent particles.

The findings indicate that the inhibitor molecules’ electronic and adsorption characteristics are highly influenced by external factors. This means that understanding the behavior of these molecules requires a multifaceted approach that considers both intrinsic molecular properties and the broader chemical context, including interactions with metal surfaces and solvents (a conclusion that further strengthens the limitation of global reactivity descriptors in effectively predicting the corrosion inhibition performance of molecules [63]).

## 4. Conclusions

This study provides an intricate understanding of the interactions between hydroxytyrosol and tyrosol (two phenolic compounds) and the iron surface, thereby underscoring their potential as effective corrosion inhibitors. Our research indicates that these compounds manifest notable adsorption characteristics onto iron surfaces coupled with substantial reactivity, attributes that can be traced back to their unique structural and electronic properties. In addition, the investigative approach, which incorporates molecular dynamics, SCC-DFTB, and quantum chemical calculations, provides comprehensive details at both the macroscopic and microscopic levels. Crucially, the insights obtained from this study highlight the potential utility of these compounds in corrosion mitigation strategies. While this study provides insights into hydroxytyrosol and tyrosol interactions with iron surfaces, some limitations exist. The scope of the simulations may not cover all experimental variables, suggesting the need for more diverse conditions in future research. Further exploration of the complex interplay between these compounds and different metal surfaces and corrosive environments as well as experimental validation may enhance our understanding. These areas present promising avenues for subsequent investigations.

## Figures and Tables

**Table 1:** Quantum chemical parameters of HTR and TRS molecules obtained by DFT at GGA/DNP theoretical level.

Molecule	E_HOMO_	E_LUMO_	∆E	IE	EA	Ƞ	χ	ω	σ	∆N	ε	ω+	ω−
HTR	−6.22	−2.116	4.104	6.22	2.116	2.052	4.168	4.232	0.487	0.158	0.236	0.545	4.713
TRS	−6.733	−6.355	0.378	6.733	6.355	0.189	6.544	113.29	5.291	−4.560	0.008	53.420	59.964

**Table 2:** Adsorption energies of HTR and TRS on Fe(110) surface obtained from molecular dynamics simulation at aqueous and vacuum phases at 303K.

Molecule	Adsorption Energy (in eV)	
	Aqueous Phase	Vacuum Phase
HTR	−4.085	−5.214
TRS	−3.803	−4.254

## Data Availability

The data presented in this study are part of ongoing studies and cannot be shared at this time.
